# Multiple-response regression analysis links magnetic resonance imaging features to de-regulated protein expression and pathway activity in lower grade glioma

**DOI:** 10.18632/oncoscience.353

**Published:** 2017-06-23

**Authors:** Michael Lehrer, Anindya Bhadra, Visweswaran Ravikumar, James Y. Chen, Max Wintermark, Scott N. Hwang, Chad A. Holder, Erich P. Huang, Brenda Fevrier-Sullivan, John B. Freymann, Arvind Rao, TCGA Glioma Phenotype Research Group

**Affiliations:** ^1^ Department of Bioinformatics and Computational Biology, University of Texas MD Anderson Cancer Center, Houston, TX, USA; ^2^ Department of Statistics, Purdue University, West Lafayette, IN, USA; ^3^ Department of Radiology, Neuroradiology Division, Stanford University, Palo Alto, CA, USA; ^4^ Diagnostic Imaging, St. Jude Children's Research Hospital, Memphis, TN, USA; ^5^ Department of Radiology and Imaging Sciences, Division of Neuroradiology, Emory University School of Medicine, Atlanta, GA, USA; ^6^ University of California San Diego Health System, San Diego, CA, USA; ^7^ Department of Radiology, San Diego VA Medical Center, San Diego, CA, USA; ^8^ Division of Cancer Treatment and Diagnosis, National Cancer Institute, Bethesda, MD, USA; ^9^ Clinical Monitoring Research Program, Leidos Biomedical Research Inc., Frederick National Laboratory for Cancer Research, Frederick, MD, USA

**Keywords:** imaging-proteomics analysis, radiomics, lower grade glioma, signaling pathway activity, multiple-response regression

## Abstract

**Background and Purpose:**

Lower grade gliomas (LGGs), lesions of WHO grades II and III, comprise 10-15% of primary brain tumors. In this *first-of-a-kind* study, we aim to carry out a radioproteomic characterization of LGGs using proteomics data from the TCGA and imaging data from the TCIA cohorts, to obtain an association between tumor MRI characteristics and protein measurements.

The availability of linked imaging and molecular data permits the assessment of relationships between tumor genomic/proteomic measurements with phenotypic features.

**Materials and Methods:**

Multiple-response regression of the image-derived, radiologist scored features with reverse-phase protein array (RPPA) expression levels generated correlation coefficients for each combination of image-feature and protein or phospho-protein in the RPPA dataset. Significantly-associated proteins for VASARI features were analyzed with Ingenuity Pathway Analysis software. Hierarchical clustering of the results of the pathway analysis was used to determine which feature groups were most strongly correlated with pathway activity and cellular functions.

**Results:**

The multiple-response regression approach identified multiple proteins associated with each VASARI imaging feature. VASARI features were found to be correlated with expression of IL8, PTEN, PI3K/Akt, Neuregulin, ERK/MAPK, p70S6K and EGF signaling pathways.

**Conclusion:**

Radioproteomics analysis might enable an insight into the phenotypic consequences of molecular aberrations in LGGs.

## INTRODUCTION

Lower grade gliomas (LGGs), those lesions graded II and III, comprise 10-15% of primary brain tumors [1]. A fraction of these tumors will progress to grade IV glioma, glioblastoma (GBM) [2, 3]. Treatment of LGGs includes surgical resection, radiotherapy and chemotherapy [4-6]. LGGs are classified into molecular subtypes based on alterations in TP53, isocitrate dehydrogenase (IDH) 1 or 2, telomerase reverse transcriptase (TERT), and the transcriptional regulator ATRX: the mutational status of these genes correlates with clinical responses and outcomes [9]. Despite these findings, challenges remain in the treatment of LGGs.

The complementary assessment of gliomas via imaging and molecular pathology approaches, for understanding the response to treatment and poor outcomes, has spurred the investigation of combining imaging data with molecular (genetic/genomic) information. This approach, termed radiogenomics or imaging-genomics, holds potential to improve LGG/GBM outcomes. Radiogenomics allows inference of molecular characteristics of a tumor from image features obtained via human or computational assessment of pre-operative MR [10] images. This allows study of the heterogeneity of the tumor both spatially and over time [11]. Features of GBM tumor radiology correlate with somatic mutations [12]. In a xenograft mouse model, genetic changes in the tumor resulted in radiologic effects [13]. The radiomics approach has been taken in lung [14], breast [15], prostate [16], as well as brain tumors [17-19], predicting clinical outcomes based on imaging features. Initial correlations were drawn between clinical outcomes and radiological features, and more recent efforts have combined genomic datasets to improve the predictive and prognostic value of imaging biomarkers [20, 21]. More recently, the availability of proteomics datasets permits the study of co-ordinated signaling activity underlying such phenotypic changes. Radiogenomics and Radioproteomics [22] approaches promise to aid characterization of the genotype-phenotype landscape in gliomas, coupled with understanding the molecular underpinnings of tumor-associated image features. Such paradigms also enable a whole tumor assessment of phenotypic heterogeneity as a complement to molecular heterogeneity for the characterization of disease state.

In LGGs however, the radioproteomics approach to associate proteomics measurements with radiological feature sets is yet to be investigated, although a small study of four patients showed correlations between mass spectra and enhancement in the corresponding tumor region [23]. We hypothesized that a regression-based association mining of whole tumor imaging and protein expression information would identify proteins related to tumor radiological features, tumor morphology (phenotype), and associated signal transduction activity for LGGs, while leveraging protein correlation structure (as might be observed from pathway data). *To our knowledge, this study is the first to integrate radiological features with proteomic measurements in lower grade gliomas*.

## RESULTS

Multiple-response regression of the combined imaging and protein expression datasets from 57 patients (demographics given in Table 1) revealed VASARI imaging features significantly associated with protein expression levels. These expression changes were in gene products known to be mutated in LGGs, including EGFR, TP53, IDH, FUBP1, NOTCH1, ATRX, and TERT [17].

**Table 1:** Patient demographic information

Mean Age at Diagnosis (Range)	47.2 (22 - 70)
Gender - M/F	24/33
Median Overall Survival (Months)	23.3
Median Disease-Free Survival (Months)	23.3
Oligodendroglioma/Astrocytoma/Oligoastrocytoma	25/13/18
Mean KPS (Range)	89.5 (50 - 100)

Demographics are given for the 57 patients included in this study.

### VASARI features correlate with differential protein levels

Based on multiple-response regression, associations (regression coefficients) were computed for each combination of imaging feature/molecule. Molecules (proteins and phospho-proteins) with associations that were statistically significant after correction for multiple comparisons were retained for further analysis (Supplementary Table 2). For example, tumor localization to the frontal lobes was positively correlated with abundance of MYH11, and was negatively associated with eIF4E expression. Each imaging feature was matched in this way with negatively-and positively-correlated lists of molecules from the RPPA dataset.

### Ingenuity pathway analysis

**Figure 1:**
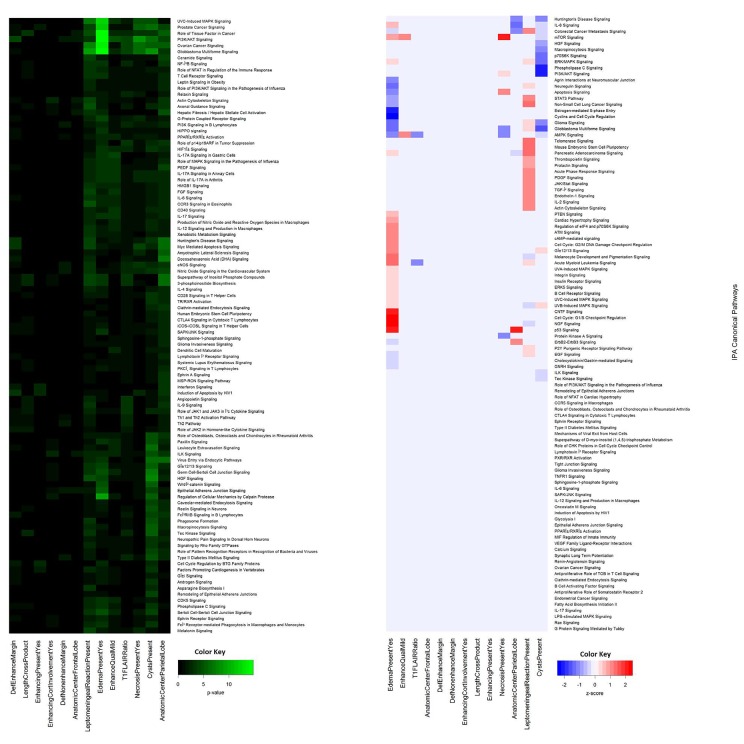
Agglomerative unsupervised hierarchical clustering of Ingenuity Pathway Analysis p-values and Z-activation scores associated with each VASARI features and IPA Canonical Pathways reveal a subset highly-correlated with pathway alterations A heat map of a representative subset of the results of the clustering of the p-values **(A)** and Z-activation scores **(B)** calculated in IPA for each imaging feature and IPA canonical pathway are shown.

**Figure 2:**
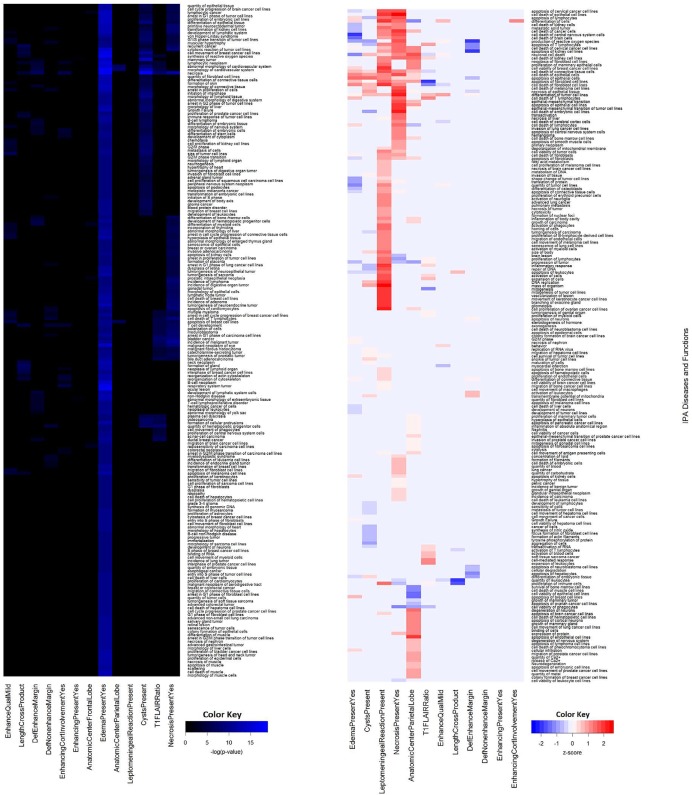
Agglomerative unsupervised hierarchical clustering of Ingenuity Pathway Analysis p-values and Z-activation scores associated with each VASARI features and IPA Diseases and Bio-functions correlated with biological dysfunction A heat map of a representative subset of the results of the clustering of the p-values **(A)** and Z-activation scores **(B)** calculated in IPA for each imaging feature and IPA diseases and bio-functions are shown.

Each VASARI feature was matched with a unique set of associated pathways and biological functions using Ingenuity Pathway Analysis software (Table 2 and Supplementary Table 3). The T1/FLAIR ratio, MRI Necrosis, leptomeningeal reaction, mild enhancement quality, edema, cysts, and tumor localization in the parietal lobe were relatively strongly associated with unique patterns of pathway activation and suppression based on their signifi correlation with unique patterns of protein expression (Table 2 and Figure 1B). Clustering analysis of the p-values associated with each VASARI feature and IPA canonical pathway revealed two groups of VASARI features (Figure 1A). Leptomeningeal reaction, edema, mild enhancement quality, cysts, T1/FLAIR ratio, MRI necrosis, and localization of anatomic center of the tumor to the parietal lobes tended to have lower p-values for each IPA canonical pathway (Figure 1A). Edema, the presence of cysts, T1/FLAIR ratio, Leptomeningial reaction, localization of tumor to Parietal lobe and MRI necrosis were most strongly associated with altered disease and biological functions (Figure 2A).

**Table 2:** Radiological features are associated with unique pathway alterations in LGG

T1/FLAIR ratio	Acute Myeloid Leukemia Signaling 6.651, Cancer Drug Resistance by Drug Efflux 5.48, AMPK Signaling 5.38
MRI Necrosis	Cancer Drug Resistance by Drug Efflux 8.968, PI3K/AKT Signaling 8.738 14-3-3-mediated Signaling 8.614
Leptomeningeal reaction	Neuregulin Signaling 15.714, Acute Myeloid Leukemia Signaling 11.297 ErbB Signaling 11.067
Cross-product length	Glucocorticoid Receptor Signaling 3.879, IL-2 Signaling 3.602, ErbB2-ErbB3 Signaling 3.537
Enhancing tumor	Molecular Mechanisms of Cancer 3.505, GADD45 Signaling 2.745, DNA damage-induced 14-3-3Ï*f* Signaling 2.745
Enhancing cortical involvement	ATM Signaling 3.408, Hereditary Breast Cancer Signaling 2.912, ILK Signaling 2.636
Mild enhancement quality	14-3-3-mediated Signaling 6.36, Aryl Hydrocarbon Receptor Signaling 6.231, AMPK Signaling 5.71
Edema	Neuregulin Signaling 15.666, Glioblastoma Multiforme Signaling 14.801, Role of Tissue Factor in Cancer 14.2
Definition of the non-enhancing margin	AMPK Signaling 3.326, Glucocorticoid Receptor Signaling 2.965, Notch signaling 2.144
Definition of the enhancing margin	VEGF Signaling 4.62, PI3K/AKT Signaling 4.379, Molecular Mechanisms of Cancer 4.369
Cysts present	Molecular Mechanisms of Cancer 8.654, Glioblastoma Multiforme Signaling 8.568, AMPK Signaling 8.046
Tumor localization in the parietal lobe	HER-2 Signaling in Breast Cancer 8.177, ErbB Signaling 7.94, Pancreatic Adenocarcinoma Signaling 7.533
Tumor localization to the frontal lobe	UVB-Induced MAPK Signaling 2.205, FLT3 Signaling in Hematopoietic Progenitor Cells 2.095, Role of Tissue Factor in Cancer 1.94

Proteins with expression significantly correlated with imaging features were analyzed by IPA. Top pathways for each feature are shown with the associated –log (p-value).

From the IPA calculation of Z-activation scores, correlations could be inferred that indicate up regulation and down regulation of a portion of IPA canonical pathways (Figure 1B), diseases, and biological functions (Figure 2B). Focusing on the strongest associations, T1/FLAIR ratio was correlated with down regulation of AMPK and acute myeloid leukemia signaling. MRI necrosis was associated with up regulation of PI3K/AKT/mTOR signaling and apoptosis, while being correlated with down regulation of AMPK and protein kinase A signaling. Leptomeningeal reaction was associated with up regulation of signaling pathways also important in pancreatic and lung cancers, while being associated with down regulation of NGF and UVB-induced MAPK signaling. Edema was implicated with increased NGF signaling, as well as increased G1/S checkpoint regulation. The presence of cysts was associated with decreased PI3K/AKT and phospholipase C signaling. Localization of tumor to the parietal lobe was correlated with upregulated p53 signaling activity, and with downregulated IL-8 signaling. These associations highlight the possible functional implications of the correlations drawn between each VASARI feature and protein expression.

## DISCUSSION

The integration of diverse biomedical datasets poses a significant analytical and computational challenge. Combining clinical, gene expression, protein, post-translational modification, and imaging datasets requires robust analytical approaches that minimize false- discovery rates and yield useful correlates between model parameters. The development of such techniques would be a major advancement in analytics and make novel diagnostics and prognostics possible [44]. Radioproteomics and radiogenomics show potential to complement proteomics and genomics for understanding the biological underpinnings of phenotypic tumor features and for predicting patient response to treatment [20]. As this approach matures, it is conceivable that imaging could provide strong surrogates to potentially replace traditional physical biopsies, allowing signaling activity status to be determined non-invasively [45].

We hypothesized that integrating imaging and protein expression data would reveal imaging biomarkers tying radiological features to the proteomics of LGG. We have demonstrated the use of a novel multiple-response regression approach to integrate imaging features and proteomics data while leveraging the correlation structure between the protein expression values. In the situation where the responses are correlated, and there are multiple predictors associated with multiple responses, standard univariate/multivariate analysis will identify associations incorrectly. Thus the correlated response regression approach is more appropriate in this context, having direct biological significance since it uses the correlation structure of the proteins during regression.

Multiple-response regression of the combined VASARI feature annotations and RPPA molecular expression data revealed significantly correlated proteins and phospho-proteins indicating upregulated signal transduction pathways. Querying the highly-correlated molecules using Ingenuity Pathway Analysis probed the biological implications of the regression results. Clustering of the pathway analysis highlighted the most important imaging features tied with underlying molecular alterations. By using this analytics pipeline, corresponding biological hypotheses about the underpinnings of these phenotypic characteristics could be within the framework of the IPA knowledge base.

Multiple-response regression revealed associations between imaging features and signal transduction, evidenced by significant correlations with levels of phosphorylated signaling molecules such as Src kinase, Yes-associated protein YAP, ribosomal protein S6, and MAPK, consistent with the established role of these molecules in tumor cell proliferation, migration and invasion. Kinase activity of over-expressed Src drives tumor cell proliferation [43]. YAP is a downstream co-activator in the Hippo pathway, a developmental mechanism frequently co-opted in tumorigenesis and progression [47]. MAPK phosphorylation participates in signaling cell growth and proliferation, and pathways which include these kinases are frequently over-active in tumor cells [48]. Ribosomal protein S6 is a component of the ribosome phosphorylated by S6 kinase, which is a component of the PI3K-mTOR axis, over-activation of which is another common feature of cancer cells [49].

In conclusion, a multiple response regression framework with correlated errors was used to assess the relationship between image-derived radiologist annotations (features) and protein measurements in TCGA LGG cases. Pathway analysis highlighted the functional effects of proteomic alterations in LGGs on MR imaging features, revealing a unique pattern of protein expression and post-translational modifications associated with each VASARI imaging feature. The downstream effects of these gene expression changes were consistent with the current understanding of tumor biology. One such example is that a physiological event, such as hemorrhage, could cause massive cell death, inducing stress response signaling in affected cells. This was reflected in the results of the pathway analysis, which associated the LGG specific protein expression correlated with hemorrhage with top canonical pathways, including MAP kinase signaling induced by ultraviolet damage. Regression and pathway analysis implicated IL8, PTEN, PI3K/Akt, Neuregulin, ERK/MAPK, p70S6K and EGF signaling pathways, mechanisms that exert control over the cell cycle, growth, and proliferation and are known to be significantly altered in gliomas [17]. These pathway alterations were most significantly associated with a subset of the VASARI features, providing a picture of the molecular underpinnings of the macroscopic radiologic features of LGGs across biological scale.

## MATERIALS AND METHODS

### TCGA low-grade glioma patient dataset

MRI data for 57 patients were obtained from The Cancer Imaging Archive (TCIA), and protein (reverse- phase protein array, RPPA) molecular data were obtained from The Cancer Genome Atlas (TCGA) data portal. These patients correspond to the TCGA/TCIA subset on whom imaging data along with matched proteomics (RPPA) information is jointly available. Clinical data were obtained from cBioPortal [24]. Institutional review board approval was not needed for this retrospective study of public TCGA data.

### MR imaging feature (phenotype) analysis

**Figure 3:**
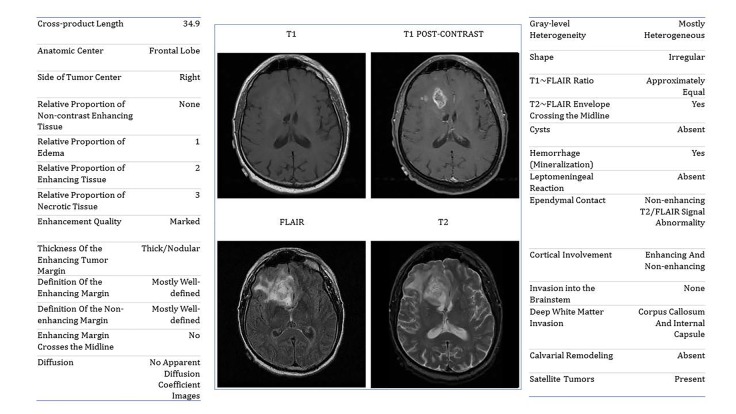
Representative axial MR T1, T1 gadolinium contrast enhanced, fluid-attenuation recovery, and T2-weighted acquisitions with corresponding VASARI scores Scores for the VASARI feature set for a representative set of MR scans are shown. MR sequences are downloaded from The Cancer Imaging Archive [47].

Adjudicated reader (radiologist) scores of the pre- operative MR images using an updated version of the standardized Visually Accessible Rembrandt Images (VASARI) feature-set were obtained from TCGA Glioma Phenotype Research Group [10]. The TCGA Glioma Phenotype Research Group readers scored cases using an updated version of the original VASARI feature-set, which was developed for assessment of GBMs, modified through expert consensus for lower grade gliomas. The imaging dataset included complete annotations for 27 of the 34 standard VASARI features. The twenty-seven VASARI features included overall shape, tumor length cross product, edema, cysts, hemorrhage (this feature can also result from mineralization), areas of brain invasion, satellite tumors, enhancement quality, thickness of the enhancing tumor margin, definition of the non-enhancing tumor margin, definition of the contrast-enhancing tumor margin, gray-level heterogeneity, enhancing cortical involvement, leptomeningeal reaction, location of the tumor anatomic center, tumor laterality to right or left cerebral hemisphere, ependymal contact, T1/FLAIR ratio, T2/FLAIR signal crossing the midline, contrast enhancement crossing the midline, calvarial remodeling, and MRI-necrosis (presumed necrosis based upon MR imaging features, as opposed to histology) (Figure 3). As indicated in Zhou et al. [25], the inter-rater analysis via kappa statistic demonstrated good inter-rater agreement along the VASARI features.

### Statistical analysis

To estimate the relationship between the LGG- VASARI imaging features and RPPA-derived protein levels, we used a multiple response regression model developed by Bhadra and Mallick [26]. The model incorporates the VASARI imaging features as the independent variables and the protein expression data (across all the RPPA proteins) as the response vector. The framework aims to capture the associations between the protein expression levels (this is biologically meaningful, since protein expression levels might be correlated), while incorporating the imaging features as covariates in the multiple predictors, multiple responses regression framework (outlined below, more details in the SI). The regression coefficient captures the association strength between the imaging predictor and the protein expression. P-values for the regression coefficients are corrected for multiple testing using Benjamini-Hochberg method [27], and statistical significance is set at 0.05.

### Multiple-response regression with correlated noise: novelty of the proposed framework

Variable selection is a ubiquitous problem in modern genomic data analysis. We provide a brief review below to explain the novelty of our approach more clearly. Further details are in the Supplementary Methods. Bayesian approaches typically perform this through an appropriate sparsity-inducing prior, e.g., spike-and-slab type prior [28, 29], double-exponential prior [30], or the more recent heavy-tailed global-local shrinkage priors such as the horseshoe [31], horseshoe+[32] and generalized double Pareto prior [33]; while frequentist approaches usually work with penalized regression models e.g., L -norm penalty of the LASSO [33], or combined l /l penalty of elastic [34], or combined L and L -norm penalty of Elastic Net [35]. However, in multiple responses regressions, such as the framework considered here, most approaches ignore the correlation structure in the error terms and treat them as independent. It is well-known that ignoring the correlation structure if the true errors are correlated leads to loss of statistical efficiency, even in low dimensions [36]. Variable selection in presence of correlated errors is being studied only recently. In frequentist settings, Rothman et al. [37] applied an algorithm based on alternating LASSO and graphical LASSO steps to simultaneously select the covariates and the error precision matrix. Yin and Li [38] applied a similar technique to high-dimensional genomic data. Cai et al. [39] designed a two-step procedure for first estimating the residual correlations and then selecting the covariates. We take a Bayesian approach and follow the framework of Bhadra and Mallick [26] in our data analysis. To control for multiple testing correction in the high- dimensional “multiple covariates, multiple responses” framework, we followed a two-stage procedure [26].

1. First we threshold the posterior probabilities of the covariates (imaging features) by controlling FDR at 0.25, yielding a sparse set of imaging predictors.
2. To determine which responses (proteomic measurements) this sparse set of covariates (imaging features) were associated with, we did another level of FDR control. We performed t-tests, of “no association” null hypothesis vs. ‘non-zero association’ alternative hypothesis, between a given imaging feature and a given protein's expression. We then declared those associations significant which had the lowest 10% of the p-values resulting from the above t-tests. All these associations are significant at the adjusted p-value of 0.05. A similar approach has previously been used successfully to analyze eQTL data [26] and glioblastoma data [41].

### Ingenuity pathway analysis

Proteins from the RPPA dataset that were found to be strongly-associated with each VASARI feature were queried using the Ingenuity Pathway Analysis software package (IPA™ QIAGEN, Redwood City, CA, http://www.qiagen.com/ingenuity). IPA Core Analyses were run on each list of mapped identifiers for each VASARI feature. In the IPA software, p-values were computed by applying the Fisher's exact test based on the number of biological functions, pathways, or molecules in the annotation as defined by the molecules in the selected Reference set, the number of molecules in the Reference set known to be associated with that function, the number of functions, pathways, and molecules in the Reference set, and the number of molecules in the Reference set [42].

### Hierarchical clustering analysis of imaging-proteomic associations

To interpret VASARI associations with pathways, diseases and functions, agglomerative unsupervised hierarchical clustering was performed on the p-values associated with each VASARI feature using the “stats” package in R. Distance matrix calculations were performed using the Euclidean method, and within-cluster variance was minimized using Ward's method [43].

## SUPPLEMENTARY MATERIALS AND METHODS TABLES



## Abbreviations

GBM: Glioblastoma
LGG: Lower grade glioma
WHO: World Health Organization
IPA: Ingenuity Pathway Analysis
VASARI: Visually Accessible Rembrandt Images
TCGA: The Cancer Genome Atlas
TCIA: The Cancer Imaging Archive
RPPA: Reverse Phase Protein Array
IDH: Isocitrate Dehydrogenase
TERT: Telomerase Reverse Transcriptase
