# Supplementary material for: Multiple-response regression analysis links magnetic resonance imaging features to de-regulated protein expression and pathway activity in lower grade glioma
Source: Oncoscience. 2017 Jun 23;4(5-6):57–66. doi: 10.18632/oncoscience.353 (PMC5538849; doi:10.18632/oncoscience.353)
Supplement: Supplementary file 1 [file oncoscience-04-0057-s001.pdf]

Multiple-response regression analysis links magnetic resonance imaging features to de-regulated protein expression and pathway activity in lower grade glioma

SUPPLEMENTARY MATERIALS AND METHODS

Model formulation

Consider a regression model of the following form: $\mathbf{Y} = \mathbf{XB} + \mathbf{E}$, where $\mathbf{Y}$ is an n × q matrix of responses, $\mathbf{X}$ is an n × p matrix of predictors, $\mathbf{E}$ is an n × q matrix of regression errors and $\mathbf{B}$ is a $p \times q$ matrix of regression coefficients. Using the notation of Dawid (1981), we further assume E follows the matrix normal distribution $\mathbf{MN}_{n\times q}(\mathbf{0}_{n\times q}, \mathbf{I}_n, \Sigma_{\mathbf{G}})$, where $\mathbf{0}_{n\times q}$ is an $n \times q$ matrix of zeros, Σ_G is the $q \times q$ covariance matrix of q possibly correlated responses and I_n is an identity matrix of size n. We assume a separable covariance structure of $\mathbf{E}$ along the rows and columns and the matrix normal formulation gives Vec($\mathbf{E}$) ~ $\mathbf{N}_{nq}(0_{nq}, \mathbf{I}_n \otimes \Sigma_{\mathbf{G}})$, a multivariate normal, with ⊗ denoting the Kronecker product. Specifically, our assumption is that the n samples are independent, but within each sample, the q responses share a common covariance structure encoded by $\Sigma_{\mathbf{G}}$. Conditional independence is modeled through an underlying (undirected) graph $\mathbf{G} = (V, \mathbf{E})$, where V corresponds to response variables $Y_1, \ldots, Y_q$, with the implication that $\{u, v\} \notin \mathbf{E} \Leftrightarrow \Sigma_G^{-1}(u, v) = 0$, implying conditional independence of u and v given the rest, where $u, v \in V$. Clearly, when p and q are much larger than n, the model is not identifiable. Thus, following Bhadra and Mallick (30) and Feldman et al. (31), we now consider a sparse formulation:

$$\mathbf{Y} = \mathbf{X}_\gamma \mathbf{B}_\gamma + \mathbf{E} \qquad (1).$$

Let $\mathbf{X}_\gamma$ be an n × p_γ is a matrix of relevant predictors encoded by the vector $\gamma = (\gamma_1, \ldots, \gamma_p) \in \{0, 1\}^p$ with $\gamma_i = 1$ if i[th] predictor is present in the model and $\gamma_i = 0$ otherwise. Thus, $p_\gamma = \sum_{i=1}^{p} \gamma_i$ is the number of active covariates. We form $\mathbf{X}_\gamma$ by dropping $(p - p_\gamma)$ columns corresponding to inactive predictors from the n × p matrix X; correspondingly, $\mathbf{B}_\gamma$ is now a $p_\gamma \times q$ matrix of regression coefficients for the selected features. E is distributed according to $\mathbf{MN}_{n\times q}(0_{n\times q}, I_n, \Sigma_{\mathbf{G}})$, as before. We consider the following hierarchical Bayesian model:

$$\square \underset{i}{\overset{i.i.d}{\sim}} \text{Bernoulli}(w_{\square}) \text{ for } i = 1, \ldots, p;\ G_{uv} \overset{i.i.d.}{\sim} P\mathbf{G}(\bullet \mid \mathbf{W}) \qquad (2)$$

$$\Sigma_{\mathbf{G}} \mid \mathbf{G} \sim \text{HIW}_G(b, d\mathbf{I}_q)\ ;\ \mathbf{B}_\gamma \mid \gamma, \Sigma_{\mathbf{G}} \sim \mathbf{MN}_{p\gamma \times q}\ \mathbf{0}_{p\gamma \times q}, c\mathbf{I}_{p\gamma}, \Sigma_G \qquad (3)$$

$$\mathbf{Y} \mid \mathbf{X}_\gamma, \mathbf{B}_\gamma, \Sigma_{\mathbf{G}} \sim \text{MN}_{n\times q}(\mathbf{X}_\gamma \mathbf{B}_\gamma, \mathbf{I}_n, \Sigma_{\mathbf{G}}) \qquad (4)$$

In Equation (2), we restrict the set of permitted graphs to the set of all decomposable graphs with nodes V, and define a prior distribution with that support as:

$$p(G \mid W) \propto \left(\prod_{\{u,v\} \in E} w_{uv} \right) \left(\prod_{\{u,v\} \notin E} (1 - w_{uv}) \right) \qquad (5)$$

The hyper-inverse Wishart (HIW) prior is conjugate for the covariance matrix in a decomposable Gaussian graphical model (Dawid and Lauritzen, 1993). Here b, c, d are fixed, positive hyper-parameters. A symmetric matrix of parameters $W = (w_{uv})_{u,\ v \in V}$ and w_γ are fixed prior probabilities, presumably close to zero, that control the sparsity in G and γ respectively. Thus, the model specifies that the priors on Σ_G and B_γ are conjugate in a graphical setting, which allows analytic marginalization of these parameters. Table S1 gives a summary of the variables used.

A collapsed gibbs sampler

Bhadra and Mallick (2013) demonstrated that one of the main advantages of the model above is that it allows a collapsed Gibbs sampler for the dependence structure G and a subset of features γ after analytically integrating out nuisance terms B_γ and Σ_G. The marginal data distribution

$$\mathbf{T}_\gamma = \mathbf{AY}, \text{ where } \mathbf{AA}^t = \mathbf{I}_n + c\ \mathbf{X}_\gamma \mathbf{X}_\gamma^t{}^{-1} \qquad (6)$$

summarizes the contributions of X and Y to the model. The hierarchical model collapses to:

$$\mathbf{T}_\gamma \mid \gamma, \mathbf{G} \sim \text{HMT}_{n\times q}(b, \mathbf{I}_n, d\mathbf{I}_q)$$

If the graphs $\mathbf{G}$ are *decomposable*, the distribution of $\mathbf{T}_\gamma \mid \gamma$, $\mathbf{G}$ is *hyper-matrix t* (33), a special type of t-distribution

which, given the graph, splits into products and ratios over the cliques and separators of the graph. We recall that a decomposable graph **G** admits a (perfect) sequence of maximal cliques $C_1, \ldots, C_k$ so that $S_j = (C_1 \cup \cdots \cup C_{j-1}) \cap C_j$, $j = 2, \ldots, k$ (called *separators*) are complete sub-graphs of **G** (33). The density of the hyper-matrix-*t* distribution $\mathrm{HMT}_{n\times q}(b, \mathbf{I}_n, d\mathbf{I}_q)$ at $\mathbf{T}_\gamma = \mathbf{t}$ is

$$f(\mathbf{t} \mid \gamma, \mathbf{G}) = \frac{\prod_{j=1}^{k} f(\mathbf{t}_{C_j} \mid \gamma, \mathbf{G})}{\prod_{j=2}^{k} f(\mathbf{t}_{S_j} \mid \gamma, \mathbf{G})}, \qquad (7)$$

$$f(\mathbf{t}_{C_j} \mid \gamma, \mathbf{G}) \propto det\left(\mathbf{I}_{|C_j|} + \mathbf{t}^t_{C_j}\mathbf{t}_{C_j} / d\right)^{-(b+n+|C_j|-1)/2}$$

and $\mathbf{t}_A$ is a $n\times|A|$ sub-matrix of **t** with columns corresponding to cliques $A \subseteq V$ in **G** (Equation (45) in Dawid and Lauritzen, 1993). The densities on the separators are defined similarly. This collapsed Gibbs sampler alleviates the need to sample $\mathbf{B}_\gamma$ and $\mathbf{\Sigma}_\mathbf{G}$ in MCMC and allows for crucial computational advantages for scaling to high dimensions and faster mixing.

MCMC algorithm

We outline the MCMC sampler algorithm of Bhadra and Mallick (2013) below and refer the interested reader to that article for details. We also follow their recommendation for the choice of hyper-parameters b, c and d.

Updating γ given G and T_γ

Searching the feature space γ is done through addition or deletion of single features. Using w_γ ~ Uniform (0, 1) gives $p(\gamma) \propto \left\{(p+1)\binom{p}{p_\gamma}\right\}^{-1}$ as prior on $\boldsymbol{\gamma}$ after integrating out w_γ with $p_\gamma = \sum_{i=1}^{p}\gamma_i$.

1. Given current set of features $\boldsymbol{\gamma}$, propose candidate $\boldsymbol{\gamma}^*$ by either (a) changing a non-zero entry in $\boldsymbol{\gamma}$ to zero with probability $(1 - \alpha_\gamma)$ and set $q(\boldsymbol{\gamma} \mid \boldsymbol{\gamma}^*) / q(\boldsymbol{\gamma}^* \mid \boldsymbol{\gamma}) = \alpha_\gamma / (1 - \alpha_\gamma)$, or (b) changing a zero entry in $\boldsymbol{\gamma}$ to one, with probability α_γ and set $q(\boldsymbol{\gamma} \mid \boldsymbol{\gamma}^*) / q(\boldsymbol{\gamma}^* \mid \boldsymbol{\gamma}) = (1 - \alpha_\gamma) / \alpha_\gamma$.
2. Calculate the likelihood $f(\mathbf{t}^* \mid \boldsymbol{\gamma}^*, \mathbf{G})$ and $f(\mathbf{t} \mid \boldsymbol{\gamma}, \mathbf{G})$ where f denotes the HMT density of Equation (7).
3. Accept the candidate γ^* with probability

$$r(\gamma, \gamma^*) = min\left\{1, \frac{f(t^* \mid \gamma^*, \mathbf{G})\, p(\gamma^*)\, q(\gamma \mid \gamma^*)}{f(t \mid \gamma, \mathbf{G})\, p(\gamma)\, q(\gamma^* \mid \gamma)}\right\}.$$

Updating G given γ and T_γ

Similar to γ; G is searched by random addition or deletion of off-diagonal edges. Using w_{uv} ~ Uniform(0, 1) and integrating out w_{uv} gives

$$\mathrm{p}(\mathrm{G}) \propto \left\{ \{q(q+1)/2\} \binom{q(q+1)/2}{r_G} \right\}^{-1}$$

where r_G is half the number of edges in the symmetric graph G. Two additional constraints for searching G are: (a) the proposed candidate G* must be decomposable. If not, propose again (a rejection scheme) and (b) the proposed candidate G* must be symmetric, since it encodes an MRF (Markov Random Field).

Ingenuity pathway analysis

Significantly-associated proteins from the RPPA dataset correlated with each VASARI feature were queried using the Ingenuity Pathway Analysis software package (IPA™ QIAGEN, Redwood City, CA, http://www.qiagen.com/ingenuity). Correlation co-efficients computed from the high-dimensional regression were used as a surrogate for fold-change. IPA Core Analyses were run on each list of mapped identifiers for each VASARI feature. In the IPA software, p-values were computed by applying the right-tailed Fisher's exact test based on the number of functions/pathways/molecules in the annotation as defined by the molecules in the selected Reference set, the number of molecules in the Reference set known to be associated with that function, the number of functions/pathways/molecules in the Reference set, and the number of molecules in the Reference set (34).

Supplementary Table 1: Description of variables and parameters comprising the multiple response regression model

Symbol	Dimension	Description	Symbol	Dimension	Description
n	scalar	sample size	$\mathbf{G}$	$q \times q$	conditional independence graph
p	scalar	number of predictor variables	$\mathbf{B}_\gamma$	$p\gamma \times q$	matrix of regression coefficients
q	scalar	number of response variables	$\mathbf{Y}$	$n \times q$	matrix of responses
p_γ	scalar	number of selected predictor variables	$\mathbf{E}$	$n \times q$	matrix of regression errors
$\boldsymbol{\gamma}$	p	vector of indicators for selecting predictors	$\boldsymbol{\Sigma}_\mathbf{G}$	$q \times q$	column covariance for errors
$\mathbf{X}$	$n \times p$	matrix of available predictors	$\mathbf{W}$	$q \times q$	symmetric matrix of edge weights
$\mathbf{X}_\gamma$	$n \times p_\gamma$	matrix of selected predictors	$\mathbf{T}_\gamma$	$n \times q$	matrix of marginal data distribution

Supplementary Table 2: Significantly-associated RPPA molecules

Vasari Feature	Positively-correlated	Negatively-correlated
Cross product length	Annexin-VII, PI3K-p85, PR	Annexin-1, Chk1-pS345, HER2, Lck, STAT5-alpha, YB-1-pS102
Tumor localization to the frontal lobe	MYH11	eIF4E
Tumor localization in the parietal lobe	Bax, Caveolin-1, EGFR-pY1068, EGFR-pY1173, HER2-pY1248, Myosin-IIa-pS1943, NDRG1-pT346, TAZ, TFRC, Transglutaminase, XPB1, YB-1-pS102, eIF4E	Annexin-VII, Bcl-2, Bim, HER3, IRS1, PI3K-p110-alpha
Edema	4E-BP1-pT70, B-Raf, Beclin, Dvl3, INPP4B, MEK1, MIG-6, N-Ras, PDK1, PDK1-pS241, PKC-pan-BetaII-pS660, PTEN, Rb-pS807-S811, SCD1, p21, p27, p53, p70S6K-pT389, p90RSK	AR, Annexin-1, Collagen-VI, Cyclin-B1, Cyclin-D1, EGFR-pY1068, Fibronectin, HER2, HER2-pY1248, Lck, MAPK-pT202-Y204, N-Cadherin, PRDX1, PREX1, S6-pS240-S244, Smad1, Src, Src-pY416, TAZ, TFRC, YAP, YAP-pS127, eIF4E, p38-MAPK, p38-pT180-Y182
Enhancement	-	Bak, Cyclin-E2
MRI Necrosis	BRCA2, Bid, ER-alpha, HSP70, Mre11, PDCD4, RBM15, XRCC1, eEF2K, p27, p27-pT198, p53, p90RSK-pT359-S363	14-3-3-zeta, AMPK-alpha, Akt, B-Raf, GAPDH, GSK3-alpha-beta, LKB1, MEK1, PDK1, PTEN, TSC1
Mild Enhancement Quality	14-3-3-zeta, ERK2, GSK3-alpha-beta-pS21-S9, GSK3-pS9, MAPK-pT202-Y204, PEA15-pS116, PRAS40-pT246, Rictor-pT1135, Transglutaminase, Tuberin-pT1462, p38-pT180-Y182	Chk2-pT68, ER-alpha, FoxM1
Definition of the enhancing margin	Bcl-xL, HSP70, β-Catenin	14-3-3-epsilon, ADAR1, Bak, CD31, Cyclin-E2, GATA3, HER3, NDRG1-pT346, Rab25, Shc-pY317
Definition of the non-enhancing margin		AMPK-pT172, FOXO3a, Notch1, p62-LCK-ligand
T1/FLAIR Ratio	BRCA2, NF2, PI3K-p110-alpha, TTF1	AMPK-pT172, B-Raf, FOXO3a, HER2, STAT5-alpha, TSC1, VHL, c-Kit, eIF4G
Cysts	4E-BP1, 53BP1, ACC-pS79, ASNS, Bap1-c-4, Caveolin-1, Cyclin-E1, Dvl3, FASN, IRS1, JNK2, Ku80, TTF1, VHL, XRCC1	ACVRL1, CD49b, Chk2, Cyclin-D1, DJ-1, N-Cadherin, PKC-delta-pS664, PRAS40-pT246, PRDX1, PREX1, SF2, Src-pY416, Src-pY527, Syk, XPB1, YAP, YAP-pS127, α-Catenin, c-Met-pY1235, mTOR-pS2448
Leptomeningeal Reaction	ASNS, ATM, BRCA2, Bid, C-Raf, Cyclin-B1, EGFR, EGFR-pY1068, EGFR-pY1173, Fibronectin, HER2-pY1248, HSP70, IGFBP2, MIG-6, PAI-1, STAT5-alpha, Smad1, c-Myc	Acetyl-a-Tubulin-Lys40, Chk1-pS345, MEK1, MEK1-pS217-S221, PEA15, PKC-delta-pS664, c-Kit, p70S6K-pT389
Enhancing Cortex Involvement	Annexin-1, Cyclin-B1, Paxillin, Rad50	MYH11, PEA15, Raptor, c-Kit

Multiple-response regression was applied to the combined VASARI feature set and RPPA dataset from 57 patients, and the results were filtered to include only molecules significantly correlated with each VASARI feature.

Supplementary Table 3: Radiological features are associated with unique biological functions in LGG

VASARI Feature	Positively-correlated Diseases and Bio-Functions	Negatively-correlated Diseases and Bio-Functions
T1/FLAIR ratio	Quantity of hematopoietic progenitor cells 1.91 Synthesis of reactive oxygen species 1.264 Cell death of T lymphocytes 1.159	Colony formation of cells -2.433 Development of genitourinary system -2.127 Development of reproductive system -2.127
MRI Necrosis	Cell death of epithelial cell lines 2.401 Differentiation of tumor cell lines 2.21 Cell death of embryonic cell lines 2.2	Cell viability of lymphocytes -2.177 Cell viability of leukocytes -2.008 Cell viability of blood cells -2.404
Leptomeningeal reaction	Cell proliferation of fibroblasts 2.77 Mass of organism 2.613 Proliferation of connective tissue cells 2.618	Apoptosis of prostate cancer cell lines -2.232 Radiosensitivity -1.974 Cell death of tumor cell lines -1.741
Cross-product length	Organismal death 1.804 Apoptosis of tumor cell lines 1.297 Apoptosis of breast cancer cell lines 1.292	Proliferation of cells -2.227 Proliferation of tumor cell lines -2 Quantity of leukocytes -1.982
Enhancing cortical involvement	Cell death of immune cells 1.972 Apoptosis 1.872 Proliferation of cells 1.53	Quantity of cells -0.89 Migration of cells -0.586 Cell proliferation of tumor cell lines -0.52
Mild enhancement quality	Cell viability of leukocytes 1.982 Senescence of fibroblast cell lines 1.953 Cell spreading 1.964	Apoptosis of carcinoma cell lines -2.433 Organismal death -1.667 Proliferation of epithelial cells -1.513
Edema	Cytostasis 2.206 Senescence of fibroblast cell lines 2.021 Radiosensitivity of carcinoma cell lines 1.982	Proliferation of tumor cells -3.114 Chemotaxis -3.1 Migration of cells -3.097
Definition of the non-enhancing margin	Quantity of cells 0.562	Cellular homeostasis -1.78 Cell viability -1.79 Expression of RNA -1.683
Definition of the enhancing margin	Organismal death 2.095 Survival of organism 1.375 Cell viability 1.314	Anoikis -1.969 Apoptosis of kidney cell lines -1.963 Production of reactive oxygen species -1.966
Cysts present	Differentiation of stem cells 1.802 Apoptosis of tumor cells 1.772 Formation of focal adhesions 1.732	Invasion of cells -2.95 Invasion of tumor cell lines -2.892 Cell movement of tumor cell lines -2.871
Tumor localization in the parietal lobe	Apoptosis of endothelial cell lines 2 neuronal cell death 1.828 Migration of breast cancer cell lines 1.725	Cellular homeostasis -1.525 Apoptosis of breast cell lines -1.452 Cell viability of epithelial cell lines -1.342

Proteins with expression significantly correlated with imaging features were analyzed by IPA. Top diseases and bio-functions for each feature are shown with the associated –log (Z-score).
